# The P2X7 Receptor-Interleukin-1 Liaison

**DOI:** 10.3389/fphar.2017.00123

**Published:** 2017-03-16

**Authors:** Anna Lisa Giuliani, Alba C. Sarti, Simonetta Falzoni, Francesco Di Virgilio

**Affiliations:** Department of Morphology, Surgery and Experimental Medicine, University of FerraraFerrara, Italy

**Keywords:** interleukin-1β, P2X7 receptor, NLRP3 inflammasome, caspase-1, inflammation

## Abstract

Interleukin-1β (IL-1β) plays a central role in stimulation of innate immune system and inflammation and in several chronic inflammatory diseases. These include rare hereditary conditions, e.g., auto-inflammatory syndromes, as well as common pathologies, such as type II diabetes, gout and atherosclerosis. A better understanding of IL-1β synthesis and release is particularly relevant for the design of novel anti-inflammatory drugs. One of the molecules mainly involved in IL-1β maturation is the P2X7 receptor (P2X7R), an ATP-gated ion channel that chiefly acts through the recruitment of the NLRP3 inflammasome-caspase-1 complex. In this review, we will summarize evidence supporting the key role of the P2X7R in IL-1β production, with special emphasis on the mechanism of release, a process that is still a matter of controversy. Four different models have been proposed: (i) exocytosis via secretory lysosomes, (ii) microvesicles shedding from plasma membrane, (iii) release of exosomes, and (iv) passive efflux across a leaky plasma membrane during pyroptotic cell death. All these models involve the P2X7R.

## The Inflammatory Process

Inflammation has been the object of countless studies and experimental observations since its definition more than 2000 years ago (Celso, De Medicina, 47 CE). Nevertheless, many aspects of this process are not fully understood, and therefore inflammation is still nowadays a field of extensive investigation, especially in view of its crucial role in the pathogenesis of many acute and chronic diseases. Accordingly, inflammation is a fertile ground of research for the development of novel drugs. Diverse chemical mediators with pro- or anti-inflammatory activity have been identified over the years. These range from histamine to bioactive lipids, e.g., prostaglandins and leukotriens, from free radicals, e.g., reactive oxygen species (ROS) and nitric oxide (NO), to cytokines, e.g., interleukins (ILs) and tumour necrosis factor (TNF). Among all these mediators, interleukin-1β (IL-1β) is recognized as one of the earliest and most potent pro-inflammatory agents synthesized and released in response to infectious agents and injuries, and therefore central to both septic and sterile inflammation (Gabay et al., 2010; Dinarello, 2011).

## An Overview on Interleukin-1 (Il-1)

The term Interleukin-1 (IL-1), also known as leukocyte endogenous mediator, hematopoietin 1, endogenous pyrogen, catabolin and osteoclast activating factor, was used in the past to indicate a factor mediating many different pro-inflammatory and catabolic effects.

The history of IL-1 begins with studies on the endogenous factor produced by activated leukocytes that causes fever. As such, IL-1 was originally described in Menkin (1943), who reported the isolation of a pyrogenic euglobulin from inflammatory exudate named “pyrexin” or “endogenous pyrogen.”

These initial studies were followed by the groundbreaking contributions of Beeson (1948) who confirmed Menkin’s observation and further reported that an endotoxin-free, protein-containing material, released from rabbit peritoneal leukocytes, caused the rapid onset of fever after injection into rabbits. This was the first time in which the mechanism behind fever, in the absence of infection, was described. After Beeson’s paper, there was a surge of studies on the links between infection/inflammation and fever, that culminated in the demonstration by Bodel and Atkins (1967) that human blood monocytes produced a pyrogen, similar to that released by rabbit neutrophils, by *de novo* synthesis.

Gery and Waksman (1972) described the effect on lymphocyte proliferation of soluble factors released in response to antigenic or mitogenic stimuli, and a few years later Dinarello and Bernheim (1981) purified the human leukocytic pyrogen from peripheral blood mononuclear cells (PBMCs) *in vitro* stimulated with heat-killed *Staphylococcus epidermidis*. Leukocytic pyrogen was also shown to enhance T cells responses to antigens and to promote synthesis of acute phase proteins (Kampschmidt et al., 1973).

Initially, the vast number of biological activities attached to a single molecule generated some confusion in the scientific community, however, with the cloning of IL-1 by Lomedico et al. (1984), the use of recombinant IL-1 established that IL-1 was indeed a pleiotropic cytokine mediating a great variety of inflammatory, as well as immunological, responses. Thanks to the seminal work of Dinarello, we now know that IL-1 is the founding member of a family of cytokines.

The IL-1 cytokine family consists of 11 members with different roles in inflammation. Seven of them, i.e., IL-1α, IL-1β, IL-18, IL-33, IL-36α, IL-36β and IL-36γ, own well-demonstrated pro-inflammatory properties, whereas four members, IL-1Ra, IL-36Ra, IL-37 and IL-38, are anti-inflammatory (Garlanda et al., 2013; Borthwick, 2016). Cytokines of the IL-1 family ligate and activate specific plasma membrane receptors, the IL-1 receptor family, comprised of 10 members, named IL-1R1, IL-1R2, IL-1R3 (IL-1RAcP), IL-R4 (ST2), IL-1R5 (IL-18Rα), IL-1R6 (IL-R36), IL-1R7 (IL-18Rβ), IL-1R8 (SIGIRR: single Ig IL-1R-related molecule or TIR8: three Ig domain-containing IL-1R related), IL-1R9 (IL-33R), IL-1R10 (TIGIRR-1) (Garlanda et al., 2013).

Interleukin-1β (IL-1β), a crucial factor of host defense in response to infections and injuries, is the best characterized and most extensively studied member of the IL-1 family (Dinarello, 1996). In the last decade, IL-1β has also emerged as a causative agent and a therapeutic target for an expanding number of systemic and local inflammatory conditions named “auto-inflammatory diseases.” The auto-inflammatory diseases include rare hereditary conditions as well as common pathologies. Recently, increasing evidence shows that the same pathogenetic mechanisms responsible for the activation of innate immunity in inherited auto-inflammatory diseases may also play a key role in sustaining inflammation in several frequent multifactorial pathologies, such as type II diabetes, gout, pseudogout, and atherosclerosis (Ginaldi et al., 2005).

Il-1β, usually not expressed by healthy resting cells, is mainly produced by activated inflammatory cells of the myeloid lineage. Production of IL-1β is a multistep process involving synthesis of immature pro-IL-1β, proteolytic cleavage to mature IL-β and, finally, release into the extracellular environment. Synthesis of the immature full-length pro-IL-1β is started with the recognition via Toll-like receptors (TLRs) of molecules derived from invading micro-organisms [pathogen-associated molecular patterns (PAMPs)] (Janeway, 2001). Once synthesized, the 31 kD pro-IL-1β undergoes a proteolytic cleavage catalyzed by caspase-1 (casp-1) which removes 116 N-terminal aminoacids to generate the 17 kD bioactive form, now ready to be secreted. If conversion to the 17 kD form does not occur, pro-IL-1β is polyubiquitinated and targeted for proteasomal degradation (Ainscough et al., 2014). Activation of casp-1, in turn, depends on assembly and activation of inflammasomes, multisubunit organelles that convert pro-casp-1 to active casp-1 (Thornberry et al., 1992; Martinon et al., 2002; Ogura et al., 2006).

The NLRP3 inflammasome has been investigated in depth and recognized as a very, likely the most, efficient machinery for pro-IL-1β maturation, and the biology of this cytokine has been intimately intertwined with that of the inflammasomes and of inflammasome-activating agents (Martinon et al., 2002; Di Virgilio, 2013). Inflammasomes are high molecular weight protein complexes assembled in the cytosolic compartment in response to a variety of stimuli, either of exogenous (PAMPs) or endogenous [danger/damage associated molecular patterns (DAMPs)] origin. PAMPs include bacteria- as well as virus derived components, whereas DAMPs encompass different classes of molecules normally segregated inside the cells (Venereau et al., 2015). DAMPs are released in response to invasion by micro-organisms (septic inflammation) as wells as to physical, chemical, metabolic non-infectious agents (sterile inflammation) (Gallucci et al., 1999). DAMPs released in the extracellular milieu fulfill the task of alerting surrounding cells, especially of immune lineages, of an incumbent danger or a damage (Venereau et al., 2015; Nie et al., 2016). Among DAMPs, extracellular ATP and other nucleotides play an undisputed role.

Nucleotide signaling is central in IL-1β maturation and release, as well as in other immune responses, such as neutrophil and macrophage chemotaxis, intracellular microbe killing, NADPH-oxidase activation, T lymphocyte proliferation and differentiation (Di Virgilio, 1995; Bours et al., 2006; Ferrari et al., 2006; Junger, 2011; Eltzschig et al., 2012; Idzko et al., 2014; Cekic and Linden, 2016). Extracellular ATP acts at plasma membrane purinergic P2 receptors, chiefly the P2X7 receptor (P2X7R) subtype, to drive NLRP3 inflammasome activation and IL-1β processing and release (Ferrari et al., 1997). ATP is released into extracellular environment during inflammation, ischemia, hypoxia, or other harmful events, via lytic (e.g., cell necrosis) or non-lytic (e.g., exocytosis, plasma membrane channels or pores) pathways. Pathways for non-lytic ATP release include pannexins (Dahl, 2015), connexins (Evans et al., 2006), ABC transporters (Cantiello, 2001), secretory vesicles (Sneddon and Westfall, 1984; Wang et al., 2013), and the P2X7R (Pellegatti et al., 2005; Suadicani et al., 2006).

## The P2X7R

Several reports underscore the pivotal role of ATP-mediated P2X7R activation in IL-1β release from activated immune cells (monocytes, macrophages, and microglia) (Di Virgilio et al., 1998; Pelegrin et al., 2008; Sanz et al., 2009). Macrophages from genetically modified mice lacking the P2X7R, ASC or NLRP3, do not release IL-1β in response to ATP (Solle et al., 2001; Mariathasan et al., 2004, 2006). Moreover, oxidized ATP, an irreversible blocker of the P2X7R (Murgia et al., 1993) abrogates ATP-induced IL-1β release from immune cells (Ferrari et al., 1997). P2X7R stimulation also induces fast release into the cytosol of oxidized mitochondrial DNA (mitoDNA) that promotes NLRP3 inflammasome assembly by direct interaction (Nakahira et al., 2011; Shimada et al., 2012).

The P2X7R is a bi-functional ATP-gated plasma membrane ion channel that upon sustained stimulation undergoes a transition that generates a non-selective pore permeable to aqueous solutes of MW up to 900 Da (Di Virgilio, 2000). The P2X7R is widely distributed in human tissues, the highest expression being in cells of the immune and inflammatory systems, especially of the myeloid lineage (Di Virgilio, 1995, 2015; Karmakar et al., 2016). The P2X7R is the seventh, and latest to be cloned, member of the P2X receptor (P2XR) subfamily activated by an agonist concentration about 100 fold higher than the other members of the family. P2XRs are ATP-gated channels permeable to monovalent (Na^+^, K^+^) and divalent (Ca^2+^) cations formed by the assembly of the same (homo) or different (hetero) P2X subunits. Six homomeric (P2X1R-P2X5R and P2X7R) and six heteromeric (P2X1/2R, P2X1/4R, P2X1/5R, P2X2/3R, P2X2/6R, and P2X4/6R) functional P2XRs have been described so far (Dubyak, 2007; North, 2016). Among P2X subunits, the P2X7 is generally thought not to assemble with the others, and thus forming P2X7 only homomeric channels. High sequence homology of P2X7R with the P2X4R (41% identity, 71% similarity), suggests a common origin by gene duplication. Therefore, the solved crystal structure for zebrafish P2X4R (Kawate et al., 2009; Hattori and Gouaux, 2012) has been used to model the 3D conformation of the P2X7R (Jiang et al., 2013). Useful insights as to ATP binding pocket, ion permeation pathway, site of antagonist binding and interaction with allosteric modulators are also derived from the crystal structure of the panda P2X7R (Karasawa and Kawate, 2016). Further information are ensued by recent 3D resolution of the human P2X3R (Mansoor et al., 2016).

The P2X subunits are characterized by a large extracellular loop, which includes agonist- and antagonist-binding sites, two short transmembrane domains, and intracellular N- and C-termini. The P2X7R with an extended C-terminal tail of 239 aa and an overall length of 595 aa, is the largest in the P2XR family. Transmembrane domains are responsible for the interactions among subunits and the formation of the ion-permeation pathway (Hattori and Gouaux, 2012; Grimes and Young, 2015). The intracellular C-tail interacts with different intracellular molecules such as heat shock proteins (HSP), cytoskeletal components, kinases and possibly also with membrane proteins. Among these latter, pannexin-1 and connexin-43 hemichannels have been variably implicated in the formation of the P2X7R-associated large-conductance pore and therefore in P2X7R-dependent IL-1β secretion, and in the release of extracellular ATP (Pelegrin and Surprenant, 2007; Baroja-Mazo et al., 2013). P2X7R has also been found to interact directly with components of inflammasomes, such as NLRP2, ASC (apoptosis-associated speck-like protein containing a CARD) and NLRP3 (Minkiewicz et al., 2013; Franceschini et al., 2015; Salaro et al., 2016). P2X7R activation by ATP is one of the most potent stimuli for NLRP3 inflammasome activation (Mariathasan et al., 2006; Munoz-Planillo et al., 2013).

## The NLRP3 Inflammasome

Inflammasomes are cellular organelles with a fundamental role in inflammation and cell death (Martinon et al., 2002; Guo et al., 2015; Rathinam and Fitzgerald, 2016). The basic scaffold subunit is a nucleotide-binding and oligomerization domain (NOD)-like receptor (NLR) that contains a C-terminal leucine-rich repeat (LRR) domain, a central NACHT nucleotide-binding domain (NOD) and an N-terminal pyrin domain (a CARD domain in the NLRC4 inflammasome). The pyrin domain of the NLR scaffold subunit interacts with the pyrin domain of an adaptor molecule named ASC. NLR-driven ASC recruitment drives pro-casp-1 activation via CARD domains present on both ASC and pro-casp-1, thus resulting in pro-casp-1 cleavage and casp-1 activation. Casp-1 then cleaves pro-IL-1β and pro-IL-18 to produce the mature forms of both cytokines (Benko et al., 2008; Schroder and Tschopp, 2010; Broz and Dixit, 2016; Prochnicki et al., 2016). Inflammasomes play a cardinal role in innate immunity thanks to their ability to sense PAMPs and DAMPs (He et al., 2016a; Kim et al., 2016). Within the subfamily of NLRP inflammasomes (i.e., inflammasomes based on NLR scaffold molecules with an N-terminal pyrin domain) NLRP3 is currently enjoying the widest popularity as crucial sensor for a large number of danger signals and as the main platform for IL-1β processing. Activating stimuli for the NLRP3 inflammasome include bacterial toxins, flagellin, muramyl dipeptide, viral nucleic acids and fungal products, as well as endogenous components such as ATP, cholesterol crystals, monosodium urate, glucose and amyloid β, environmental pollutants, such as silica, asbestos or physical agents such as UV radiations (Kim et al., 2016).

The identity of the activating stimulus of the NLRP3 inflammasome has been a hot issue ever since its discovery. Nowadays there is basically general consensus on the key role played by K^+^ efflux, which seems to be the final common pathway for many different agents (Munoz-Planillo et al., 2013). Most efficient NLRP3 activators include extracellular ATP, K^+^ ionophores, and several extracellular crystals, all known to decrease the cytosolic K^+^ level. The mechanism whereby these different agents lower K^+^ is not entirely clear, but many converge on P2X7R activation (Alves et al., 2014; Prochnicki et al., 2016). In fact, while P2X7R opening or nigericin, a carboxylic K^+^ ionophore, directly allow K^+^ efflux along its concentration gradient, the mechanism by which crystals, such as monosodium urate, deplete intracellular K^+^ is obscure. To support the contribution of K^+^ depletion, drugs inhibiting the Na^+^/K^+^-ATPase also trigger NLRP3 inflammasome activation (Walev et al., 1995; Munoz-Planillo et al., 2013). Albeit inhibition of Na^+^/K^+^-ATPase also causes plasma membrane depolarization, there is no evidence that depolarization itself may trigger P2X7R pore opening and/or IL-1β release (Di Virgilio, 2013). The central role of intracellular K^+^ is further supported by the finding that a K^+^ drop is also necessary to allow recruitment of the Nima-related kinase (NEK)7 protein to the NLRP3 inflammasome (He et al., 2016b). On the other hand, the mechanism by which the drop in the K^+^ concentration drives NEK7 recruitment, NLRP3 inflammasome assembly and activation is utterly unknown.

The NLRP3 inflammasome can be also activated by a non-canonical pathway involving casp-11. Casp-11, and its human orthologs casp-4 and -5, function as cytosolic LPS sensors (Shi et al., 2014). Once activated by LPS, casp-11 induces cleavage of the plasma membrane channel pannexin-1 (Yang et al., 2015) producing two events consisting of K^+^ efflux, that activates NLRP3, and release of ATP that acts as a P2X7R agonist to promote further NLRP3 activation and cell death (Yang et al., 2015). The casp-11/pannexin-1/NLRP3 inflammasome axis is proposed to promote IL-1β/IL-18 production (Yang et al., 2015). In addition, active casp-11 triggers pyroptosis via cleavage of Gasdermin D (GSDMD) leading to accumulation of free active N-terminal domains of this protein which disrupt cellular functions by forming plasma membrane pores (He et al., 2015; Vince and Silke, 2016). Casp-11-mediated cell death, like casp-1-induced pyroptosis, requires cleavage of the GSDMD pyroptotic factor (He et al., 2015; Kayagaki et al., 2015; Shi et al., 2015). Casp-11 mediated cell death is indeed abrogated in GSDMD deficient cells and, although it is not clear if GSDMD is the terminal pyroptotic factor, its N-terminal domain released following casp-11-dependent cleavage is sufficient to cause pyroptosis (Kayagaki et al., 2015; Shi et al., 2015). It has been proposed that, since casp-1 is required for both pyroptosis and IL-1β cleavage, IL-1β is passively released alongside DAMPs following plasma membrane rupture (Vince and Silke, 2016). The finding that in macrophages lack of GSDMD has no effect on NLRP3-stimulated IL-1β processing by casp-1 but prevents IL-1β secretion (He et al., 2015; Shi et al., 2015), suggests that casp-1 is necessary for IL-1β cleavage whereas GSDMD is indispensable for its release. Recent findings have revealed that in human monocytes stimulated with LPS casp-4 and -5 act as key determinants in one-step non-canonical NLRP3 inflammasome activation culminating with IL-1β release (Vigano et al., 2015). This one-step pathway has been suggested to require Syk activity and Ca^2+^ influx due to CD14/TLR4-mediated LPS internalization (Vigano et al., 2015). NLRP3 activation and IL-1β release can also be driven by K^+^ independent mechanisms involving ROS generation or RIPK1/FADD/casp-8 recruitment (Zhou et al., 2011; Heid et al., 2013; He et al., 2016a; Sanman et al., 2016). Converging experimental findings seem to rule out a role for cytosolic Ca^2+^ increases (Brough et al., 2003; Rada et al., 2014; Katsnelson et al., 2015). In some non-immune cells, e.g., astrocytes, IL-1β maturation has been reported to be due to P2X7-dependent NLRP2 stimulation via a process involving direct NLRP2, P2X7R, pannexin-1 interaction (Minkiewicz et al., 2013). Finally, IL-1β can also be processed independently of inflammasome/casp-1 activation, as shown in casp-1 deficient mice, where pro-IL-1β to IL-1β extracellular conversion is catalyzed by various neutrophil proteases such as elastase, proteinase-3, granzyme A and cathepsine G (Fantuzzi et al., 1997; Joosten et al., 2009).

P2X7R stimulation by itself has no or little effect on pro-IL-1β cytoplasmic accumulation, therefore cells need priming by agents that promote IL-1β gene transcription, which mainly occur via NFκB activation. Typical priming agents are bacterial lipopolysaccharide, zymosan and poly(I:C) (Ferrari et al., 1996; Facci et al., 2014).

## IL-1β Release

**FIGURE 1:**
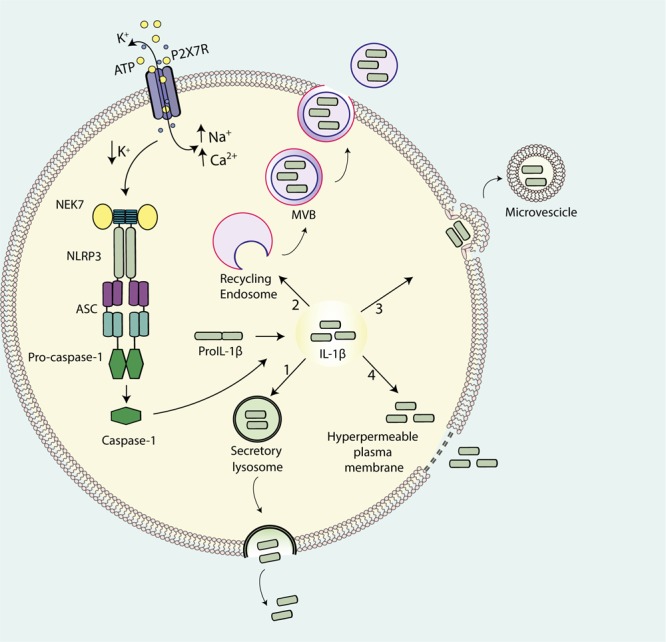
**Pathways for IL-1β release from activated immune cells.** IL-1β maturation is catalyzed by ATP-mediated stimulation of the P2X7R that drives NLRP3 inflammasome assembly and casp-1 recruitment. Four models have been proposed for IL-1β release: (1) exocytosis of secretory lysosomes; (2) shedding of plasma membrane-derived microvesicles; (3) exocytosis of multivesicular body (MVB)-derived exosomes; (4) passive efflux across hyperpermeable plasma membrane during pyroptotic cell death.

The canonical pathway for the export of cellular proteins into the extracellular space involves the ER and the Golgi apparatus that together form the endo-membrane system through which the vast majority of proteins are either targeted to the extracellular space or to specialized sub-cellular compartments. At variance with other cytokines, IL-1β lacks the conventional leader/signal peptide and therefore is not targeted to the conventional ER-Golgi secretory pathway (Rubartelli et al., 1990). This leads to IL-1β accumulation into the cytosol after translation on free ribosomes. Moreover, conversion of pro-IL-1 β to the mature form by inflammasomes also takes place in the cytosol. Therefore, release of mature IL-1 β requires non-classical mechanisms of export from the cytosolic compartment (Rubartelli et al., 1990; Wewers, 2004; Eder, 2009). A number of different possible mechanisms have been proposed (Dubyak, 2012) and summarized in **Figure 1**. They include exocytosis via secretory lysosomes (Andrei et al., 1999; Andrei et al., 2004), microvesicle shedding from plasma membrane (MacKenzie et al., 2001; Bianco et al., 2005; Pizzirani et al., 2007), release of exosomes (Qu et al., 2007), and, lastly, passive efflux across a leaky plasma membrane during pyroptotic cell death (Bergsbaken et al., 2009; Martin-Sanchez et al., 2016). The P2X7R has been implicated in all these processes.

## Exocytosis of IL-1β-Containing Secretory Lysosomes

Rubartelli et al. (1990) presented the first evidence for a non-classical secretory pathway for IL-1β release. Blockade of protein transport and secretion through the ER-Golgi complex did not affect IL-1β release, thus pointing to the involvement of secretory lysosomes. Secretory lysosomes are unusual organelles found principally in hematopoietic cells with a dual-function, degradative and secretory (Blott and Griffiths, 2002). The exocytic process can be triggered by different stimuli among which ATP, possibly via the increase in the intracellular Ca^2+^ concentration. Migration of exocytic lysosomes to the plasma membrane is a microtubule-dependent process that brings the lysosomes close to the plasma membrane allowing fusion and release of their content into the extracellular space. This model for IL-1β secretion is mainly based on morphological evidence from ATP-stimulated monocytes where IL-1β was found to be trapped within organelles akin to late endosomes and early lysosomes (Andrei et al., 1999). In human monocytes and mouse macrophages, ATP-stimulated, P2X7R-dependent release of mature IL-1β and casp-1 strongly correlated with secretion of the lysosomal markers cathepsin B, cathepsin D and lysosomal-associated membrane protein 1 (LAMP1) (Andrei et al., 1999; Carta et al., 2006). Both IL-1β and casp-1 are found in the extracellular medium 20 min after ATP stimulation, suggesting a similar time course. According to Rubartelli and coworkers a fraction of intracellular pro-IL-1β is co-stored together with pro-casp-1 within the secretory lysosomes, ready to be secreted in response to P2X7R stimulation (Rubartelli et al., 1990). The triggering stimulus is thought to be the P2X7R-induced loss of intracellular K^+^, which activates a phosphatidylcholine-specific phospholipase C, which in turn causes an increase in cytosolic Ca^2+^, Ca^2+^-dependent phospholipase A_2_ activation and finally exocytosis of the IL-1β-containing lysosomes. These events are blocked by inhibitors of phospholipase A_2_ or phosphatidylcholine-specific phospholipase C. This model suggests that, whereas the massive K^+^ efflux due to P2X7R activation has a key role in the maturation of pro-IL-1β, the intracellular Ca^2+^ increase is more directly responsible for IL-1β secretion.

## Shedding of IL-1β-Containing Plasma Membrane Microvesicles

**FIGURE 2:**
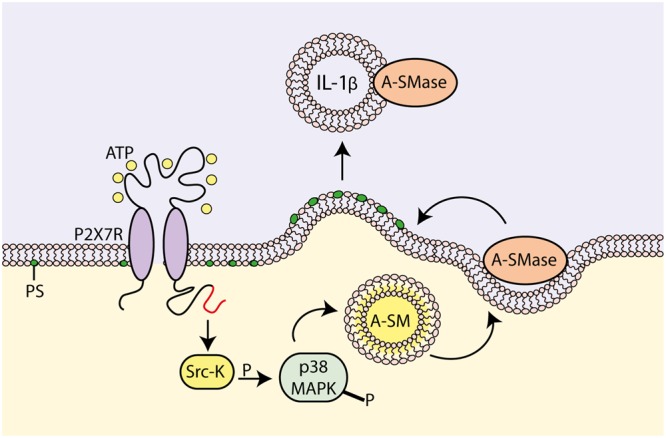
**Molecular mechanism for P2X7R-dependent microvesicle shedding.** P2X7R activation promotes interaction of the C-terminal domain with a src-protein tyrosine kinase (Srk-K), which in turn phosphorylates P38 MAP kinase (P38 MAPK). P38 MAPK induces flip of acidic sphingomyelinase (A-SMase) from the inner to the outer plasma membrane leaflet. On the outer plasma membrane leaflet, A-SMase hydrolyzes sphingomyelin to generate ceramide that in turn alters membrane fluidity, drives formation of plasma membrane blebs and promotes shedding of IL-1β-containing microvesicles (modified from Bianco et al., 2009).

Surprenant and coworkers proposed a different vesicular mechanism for IL-1β release from THP-1 monocytes (MacKenzie et al., 2001). According to this mechanism, P2X7R stimulation induces mature IL-1β accumulation at discrete sub-plasmalemmal sites, where from it is then trapped into small plasma membrane blebs that are finally rapidly shed as microvesicles into the extracellular space (**Figure 2**). Microvesicle shedding is preceded by flip of phosphatidylserine (PS) to the outer leaflet of the plasma membrane. Microvesicles size ranges from 200 nm to 1 μm, which makes them distinct from the much larger apoptotic bodies derived from apoptotic cells (1–4 μm size), and the smaller exosomes derived from intraluminal vesicles of endosomal multivesicular bodies (MVBs). A similar mechanism for IL-1β release has also been observed in human monocyte-derived dendritic cells (DCs) and mouse microglia (Bianco et al., 2005; Pizzirani et al., 2007). Shed microvesicles contain (a) plasma membrane phospholipids, e.g., PS (MacKenzie et al., 2001), (b) membrane intrinsic proteins, such as P2X7R, CD63, CD39, MHC-II, LAMP1 (Andrei et al., 1999; Pizzirani et al., 2007) and (c) cytoplasmic proteins, such as pro-IL-1β, pro-casp-1, IL-1β, casp-1, casp-3 and cathepsin D (Gudipaty et al., 2003; Andrei et al., 2004; Bianco et al., 2005; Carta et al., 2006; Pizzirani et al., 2007; Qu et al., 2007). It is not clear how and if IL-1β finally effluxes out of the microvesicles, thus fulfilling its role as an extracellular signaling molecule, or alternatively is delivered intracellularly following microvesicle fusion with the plasma membrane of target cells. Verderio and coworkers provided ample evidence showing that microvesicles released from P2X7R-stimulated microglia fuse with the plasma membrane of target cells (e.g., neurons), deliver their content and affect target cell responses (e.g., synaptic activity) (Antonucci et al., 2012; Turola et al., 2012; Verderio et al., 2012). We reported some time ago that microvesicles shed from P2X7R-stimulated DCs express the P2X7R and are lysed by exposure to extracellular ATP, thus releasing their cargo of IL-1β (Pizzirani et al., 2007). This observation led us to propose that IL-1β is released in the vicinity of the target cell plasma membrane by ATP-stimulated and P2X7R-dependent microvesicle rupture (Pizzirani et al., 2007). In fact, it is known that due to continuous ATP release into the extracellular space, cells are surrounded by an “ATP halo” that generates an ATP concentration higher in the vicinity of the plasma membrane than in the bulk solution. Thanks to this ATP gradient, microvesicle journey across the interstitial space should be relatively safe until they reach the target cell surface where they are supposed to find an ATP concentration sufficient to activate the P2X7R and trigger lysis.

## Exocytosis of IL-1β-Containing Exosomes

In mouse bone marrow-derived macrophages (BMDMs) the main mechanism for non-classical IL-1β release has been reported to be neither secretory lysosomes nor microvesicle shedding, but rather P2X7R-stimulated MVBs formation and exosome release (Qu et al., 2007). Exosomes are small vesicles (30–100 nm) released upon fusion of MVBs with the cell plasma membrane. Exosomes originate as intraluminal vesicles during the process of MVBs formation. MVBs or late endosomes are components of the endocytic pathway that range from 250 to 1000 nm in diameter. MVBs can either be degraded or fuse with the plasma membrane, releasing the intraluminal vesicles into the extracellular space. Intraluminal vesicles are then referred to as exosomes following their extracellular release. During the process of formation, transmembrane and peripheral membrane proteins are incorporated into the exosome membrane, while cytosolic components are enclosed within the vesicles. Exosomes released from macrophages, DCs or B-lymphocytes contain soluble proteins present in the cytosol, such as pro-IL-1β, pro-casp-1 and the respective mature form IL-1β and casp-1, and plasma membrane proteins such as MHCI and MHCII, a feature of exosomes derived from antigen presenting cells. From P2X7R-stimulated BMDMs two distinct types of membrane-bound vesicles are shed: (a) plasma membrane-derived microvesicles carrying P2X7R and LAMP1, and (b) MVB-derived exosomes lacking both P2X7R and LAMP1. However, both types of vesicles are able to present peptide-MHCII complexes to T cells (Ramachandra et al., 2010). Secretion of IL-1β and MHCII are strongly inhibited in mice deleted of ASC and NLRP3, suggesting the possibility that inflammasome complex regulate the formation of MVBs and the accumulation of IL-1β and casp-1, although the mechanism remains unclear (Qu et al., 2009).

## IL-1β Release as a Consequence of Plasma Membrane Damage and Cell Death

A model for IL-1β release involving plasma membrane damage and cell death (whether by necrosis or apoptosis) has been proposed several years ago (Hauser et al., 1986; Hogquist et al., 1991). A major obstacle for the acceptance of this model is the need for proteolytical activation of pro-IL-1β, which is assumed to occur coordinately with its secretion, and the consistent observation that cytoplasmic mature IL-1β levels are very low (Perregaux et al., 1992). Of course, it is possible that extracellular proteases, e.g., trypsin or cathepsins might do the job, but *in vivo* relevance of extracellular pro-IL-1β maturation is dubious. However, in a recent paper, Pelegrin and co-workers have re-visited the cell permeabilization/cell death model for IL-1β release from BMDMs taking advantage of novel, highly sensitive, fluorescence-based technique to measure IL-1β secretion and of a novel inhibitor, punicalagin (Martin-Sanchez et al., 2016). Rigorous analysis of release of the cytoplasmic marker lactic dehydrogenase and of IL-1β revealed that the kinetics of two processes were closely over-imposed. Furthermore, punicalagin, a polyphenolic compound that efficiently prevents plasma membrane permeabilization in response to a number of membrane-perturbing agents, fully abolished ATP-dependent IL-1β secretion but not its processing, thus showing that pro-IL-1β cleavage and mature IL-1β secretion can be dissociated, and that a “leaky membrane” is needed for IL-1β release. Since casp-1 activation is also a major driver of pyroptotic cell death, Pelegrin and co-workers suggested that in macrophages IL-1β secretion occurs via a non-specific increase in plasma membrane permeability associated to cell death (Martin-Sanchez et al., 2016).

## Is the P2X7R-Targeting a Therapeutically Live Option?

Several studies show that P2X7R blockade efficiently antagonize IL-1β release in different disease experimental models (Bartlett et al., 2014). However, similar evidence from human studies is lacking. Measurement of serum IL-1 in autoimmune and autoinflammatory diseases is seldom significantly elevated, and is not thought to be a reliable indicator of inflammation (Dinarello, 2005). Therefore, it is not possible to verify in humans whether P2X7R blockade has any effect on IL-1β release. Assessing the *in vivo* effect of P2X7R blockade on IL-1, and in general, all cytokines, release, is made even more complex by the disappointing results of most clinical trials so far carried out (De Marchi et al., 2016; Jacobson and Muller, 2016).

## Conclusion

Extracellular ATP is now acknowledged to be one of the earliest most ubiquitous DAMPs (Di Virgilio, 2013; Kepp et al., 2014; Hammad and Lambrecht, 2015; Venereau et al., 2015). Its remarkable efficiency and plasticity as an alarm signal strongly depends on the diverse of ATP-selective plasma membrane receptors expressed by immune cells. Very interestingly, even before all ATP receptors (P2 receptors) expressed by immune cells were cloned and fully characterized, it was clear that stimulation with extracellular ATP was able to cause a dramatic acceleration of pro-IL-1β processing and release from monocytes/macrophages, as well as from microglial cells, and this was very likely a receptor-mediated event (Perregaux and Gabel, 1994; Di Virgilio et al., 1996; Ferrari et al., 1996). About at the same time the P2X7R was cloned (Surprenant et al., 1996), and soon after identified as the molecule responsible for ATP-dependent mature IL-1β release (Ferrari et al., 1997). Thus, the association between IL-1β and the P2X7R is rock solid and long standing. However, this has not led to the introduction of any P2X7R-targeted anti-inflammatory therapy, despite large effort by virtually all major Pharma Industries. Are we missing some crucial information of P2X7R and IL-1β biology, or is there a recurrent fault in P2X7R-targeting drug design and development, or both?

## Author Contributions

FDV coordinated writing and reviewed the MS. AG wrote sections of the MS. AS wrote sections of the MS. SF wrote sections of the MS.

## Conflict of Interest Statement

FDV serves as a member of the Scientific Advisory Board of Biosceptre International Limited. The other authors declare that the research was conducted in the absence of any commercial or financial relationships that could be construed as a potential conflict of interest.

## References

[B1] Ainscough J. S. Frank Gerberick G. Zahedi-Nejad M. Lopez-Castejon G. Brough D. Kimber I. (2014). Dendritic cell IL-1alpha and IL-1beta are polyubiquitinated and degraded by the proteasome. *J. Biol. Chem.* 289 35582–35592. 10.1074/jbc.M114.595686 25371210 PMC4271241

[B2] Alves L. A. de Melo Reis R. A. de Souza C. A. de Freitas M. S. Teixeira P. C. Neto Moreira Ferreira D. (2014). The P2X7 receptor: shifting from a low- to a high-conductance channel - an enigmatic phenomenon? *Biochim. Biophys. Acta* 1838 2578–2587. 10.1016/j.bbamem.2014.05.015 24857862

[B3] Andrei C. Dazzi C. Lotti L. Torrisi M. R. Chimini G. Rubartelli A. (1999). The secretory route of the leaderless protein interleukin 1beta involves exocytosis of endolysosome-related vesicles. *Mol. Biol. Cell* 10 1463–1475. 10.1091/mbc.10.5.1463 10233156 PMC25302

[B4] Andrei C. Margiocco P. Poggi A. Lotti L. V. Torrisi M. R. Rubartelli A. (2004). Phospholipases C and A2 control lysosome-mediated IL-1 beta secretion: implications for inflammatory processes. *Proc. Natl. Acad. Sci. U.S.A.* 101 9745–9750. 10.1073/pnas.0308558101 15192144 PMC470745

[B5] Antonucci F. Turola E. Riganti L. Caleo M. Gabrielli M. Perrotta C. (2012). Microvesicles released from microglia stimulate synaptic activity via enhanced sphingolipid metabolism. *EMBO J.* 31 1231–1240. 10.1038/emboj.2011.489 22246184 PMC3297996

[B6] Baroja-Mazo A. Barbera-Cremades M. Pelegrin P. (2013). The participation of plasma membrane hemichannels to purinergic signaling. *Biochim. Biophys. Acta* 1828 79–93. 10.1016/j.bbamem.2012.01.002 22266266

[B7] Bartlett R. Stokes L. Sluyter R. (2014). The P2X7 receptor channel: recent developments and the use of P2X7 antagonists in models of disease. *Pharmacol. Rev.* 66 638–675. 10.1124/pr.113.008003 24928329

[B8] Beeson P. B. (1948). Temperature-elevating effect of a substance obtained from polymorphonuclear leucocytes. *J. Clin. Invest.* 27 524. 18939147

[B9] Benko S. Philpott D. J. Girardin S. E. (2008). The microbial and danger signals that activate Nod-like receptors. *Cytokine* 43 368–373. 10.1016/j.cyto.2008.07.013 18715799

[B10] Bergsbaken T. Fink S. L. Cookson B. T. (2009). Pyroptosis: host cell death and inflammation. *Nat. Rev. Microbiol.* 7 99–109. 10.1038/nrmicro2070 19148178 PMC2910423

[B11] Bianco F. Perrotta C. Novellino L. Francolini M. Riganti L. Menna E. (2009). Acid sphingomyelinase activity triggers microparticle release from glial cells. *EMBO J.* 28 1043–1054. 10.1038/emboj.2009.45 19300439 PMC2664656

[B12] Bianco F. Pravettoni E. Colombo A. Schenk U. Moller T. Matteoli M. (2005). Astrocyte-derived ATP induces vesicle shedding and IL-1 beta release from microglia. *J. Immunol.* 174 7268–7277. 10.4049/jimmunol.174.11.7268 15905573

[B13] Blott E. J. Griffiths G. M. (2002). Secretory lysosomes. *Nat. Rev. Mol. Cell Biol.* 3 122–131. 10.1038/nrm732 11836514

[B14] Bodel P. Atkins E. (1967). Release of endogenous pyrogen by human monocytes. *N. Engl. J. Med.* 276 1002–1008. 10.1056/NEJM196705042761803 5228572

[B15] Borthwick L. A. (2016). The IL-1 cytokine family and its role in inflammation and fibrosis in the lung. *Semin. Immunopathol.* 38 517–534. 10.1007/s00281-016-0559-z 27001429 PMC4896974

[B16] Bours M. J. Swennen E. L. Di Virgilio F. Cronstein B. N. Dagnelie P. C. (2006). Adenosine 5’-triphosphate and adenosine as endogenous signaling molecules in immunity and inflammation. *Pharmacol. Ther.* 112 358–404. 10.1016/j.pharmthera.2005.04.013 16784779

[B17] Brough D. Le Feuvre R. A. Wheeler R. D. Solovyova N. Hilfiker S. Rothwell N. J. (2003). Ca2+ stores and Ca2+ entry differentially contribute to the release of IL-1 beta and IL-1 alpha from murine macrophages. *J. Immunol.* 170 3029–3036. 10.4049/jimmunol.170.6.3029 12626557

[B18] Broz P. Dixit V. M. (2016). Inflammasomes: mechanism of assembly, regulation and signalling. *Nat. Rev. Immunol.* 16 407–420. 10.1038/nri.2016.58 27291964

[B19] Cantiello H. F. (2001). Electrodiffusional ATP movement through CFTR and other ABC transporters. *Pflugers. Arch.* 443(Suppl. 1) S22–S27. 10.1007/s004240100639 11845298

[B20] Carta S. Tassi S. Semino C. Fossati G. Mascagni P. Dinarello C. A. (2006). Histone deacetylase inhibitors prevent exocytosis of interleukin-1beta-containing secretory lysosomes: role of microtubules. *Blood* 108 1618–1626. 10.1182/blood-2006-03-014126 16684958 PMC1895509

[B21] Cekic C. Linden J. (2016). Purinergic regulation of the immune system. *Nat. Rev. Immunol.* 16 177–192. 10.1038/nri.2016.4 26922909

[B22] Dahl G. (2015). ATP release through pannexon channels. *Philos. Trans. R. Soc. Lond. B Biol. Sci.* 370:20140191 10.1098/rstb.2014.0191 PMC4455760 26009770

[B23] De Marchi E. Orioli E. Dal Ben D. Adinolfi E. (2016). P2X7 receptor as a therapeutic target. *Adv. Protein Chem. Struct. Biol.* 104 39–79. 10.1016/bs.apcsb.2015.11.004 27038372

[B24] Di Virgilio F. (1995). The P2Z purinoceptor: an intriguing role in immunity, inflammation and cell death. *Immunol. Today* 16 524–528. 10.1016/0167-5699(95)80045-X 7495489

[B25] Di Virgilio F. (2000). Dr. Jekyll/Mr. Hyde: the dual role of extracellular ATP. *J. Auton. Nerv. Syst.* 81 59–63. 10.1016/S0165-1838(00)00114-4 10869701

[B26] Di Virgilio F. (2013). The therapeutic potential of modifying inflammasomes and NOD-like receptors. *Pharmacol. Rev.* 65 872–905. 10.1124/pr.112.006171 23592611

[B27] Di Virgilio F. (2015). P2X receptors and inflammation. *Curr. Med. Chem.* 22 866–877. 10.2174/0929867322666141210155311 25524252

[B28] Di Virgilio F. Chiozzi P. Falzoni S. Ferrari D. Sanz J. M. Venketaraman V. (1998). Cytolytic P2X purinoceptors. *Cell Death Differ.* 5 191–199. 10.1038/sj.cdd.4400341 10200464

[B29] Di Virgilio F. Ferrari D. Falzoni S. Chiozzi P. Munerati M. Steinberg T. H. (1996). P2 purinoceptors in the immune system. *Ciba Found. Symp.* 198 290–302; discussion302–295. 8879832 10.1002/9780470514900.ch17

[B30] Dinarello C. A. (1996). Biologic basis for interleukin-1 in disease. *Blood* 87 2095–2147. 8630372

[B31] Dinarello C. A. (2005). Blocking IL-1 in systemic inflammation. *J. Exp. Med.* 201 1355–1359. 10.1084/jem.20050640 15867089 PMC2213199

[B32] Dinarello C. A. (2011). Interleukin-1 in the pathogenesis and treatment of inflammatory diseases. *Blood* 117 3720–3732. 10.1182/blood-2010-07-273417 21304099 PMC3083294

[B33] Dinarello C. A. Bernheim H. A. (1981). Ability of human leukocytic pyrogen to stimulate brain prostaglandin synthesis in vitro. *J. Neurochem.* 37 702–708. 10.1111/j.1471-4159.1982.tb12544.x 7276950

[B34] Dubyak G. R. (2007). Go it alone no more–P2X7 joins the society of heteromeric ATP-gated receptor channels. *Mol. Pharmacol.* 72 1402–1405. 10.1124/mol.107.042077 17895406

[B35] Dubyak G. R. (2012). P2X7 receptor regulation of non-classical secretion from immune effector cells. *Cell Microbiol.* 14 1697–1706. 10.1111/cmi.12001 22882764 PMC3473166

[B36] Eder C. (2009). Mechanisms of interleukin-1beta release. *Immunobiology* 214 543–553. 10.1016/j.imbio.2008.11.007 19250700

[B37] Eltzschig H. K. Sitkovsky M. V. Robson S. C. (2012). Purinergic signaling during inflammation. *N. Engl. J. Med.* 367 2322–2333. 10.1056/NEJMra1205750 23234515 PMC3675791

[B38] Evans W. H. De Vuyst E. Leybaert L. (2006). The gap junction cellular internet: connexin hemichannels enter the signalling limelight. *Biochem. J.* 397 1–14. 10.1042/BJ20060175 16761954 PMC1479757

[B39] Facci L. Barbierato M. Marinelli C. Argentini C. Skaper S. D. Giusti P. (2014). Toll-like receptors 2, -3 and -4 prime microglia but not astrocytes across central nervous system regions for ATP-dependent interleukin-1beta release. *Sci. Rep.* 4:6824 10.1038/srep06824 PMC5381369 25351234

[B40] Fantuzzi G. Ku G. Harding M. W. Livingston D. J. Sipe J. D. Kuida K. (1997). Response to local inflammation of IL-1 beta-converting enzyme- deficient mice. *J. Immunol.* 158 1818–1824. 9029121

[B41] Ferrari D. Chiozzi P. Falzoni S. Dal Susino M. Melchiorri L. Baricordi O. R. (1997). Extracellular ATP triggers IL-1 beta release by activating the purinergic P2Z receptor of human macrophages. *J. Immunol.* 159 1451–1458. 9233643

[B42] Ferrari D. Pizzirani C. Adinolfi E. Lemoli R. M. Curti A. Idzko M. (2006). The P2X7 receptor: a key player in IL-1 processing and release. *J. Immunol.* 176 3877–3883. 10.4049/jimmunol.176.7.3877 16547218

[B43] Ferrari D. Villalba M. Chiozzi P. Falzoni S. Ricciardi-Castagnoli P. Di Virgilio F. (1996). Mouse microglial cells express a plasma membrane pore gated by extracellular ATP. *J. Immunol.* 156 1531–1539. 8568257

[B44] Franceschini A. Capece M. Chiozzi P. Falzoni S. Sanz J. M. Sarti A. C. (2015). The P2X7 receptor directly interacts with the NLRP3 inflammasome scaffold protein. *FASEB J.* 29 2450–2461. 10.1096/fj.14-268714 25690658

[B45] Gabay C. Lamacchia C. Palmer G. (2010). IL-1 pathways in inflammation and human diseases. *Nat. Rev. Rheumatol.* 6 232–241. 10.1038/nrrheum.2010.4 20177398

[B46] Gallucci S. Lolkema M. Matzinger P. (1999). Natural adjuvants: endogenous activators of dendritic cells. *Nat. Med.* 5 1249–1255. 10.1038/15200 10545990

[B47] Garlanda C. Dinarello C. A. Mantovani A. (2013). The interleukin-1 family: back to the future. *Immunity* 39 1003–1018. 10.1016/j.immuni.2013.11.010 24332029 PMC3933951

[B48] Gery I. Waksman B. H. (1972). Potentiation of the T-lymphocyte response to mitogens. II. The cellular source of potentiating mediator(s). *J. Exp. Med.* 136 143–155. 10.1084/jem.136.1.143 5033418 PMC2139186

[B49] Ginaldi L. Di Benedetto M. C. De Martinis M. (2005). Osteoporosis, inflammation and ageing. *Immun. Ageing* 2:14 10.1186/1742-4933-2-14 PMC1308846 16271143

[B50] Grimes L. Young M. T. (2015). Purinergic P2X receptors: structural and functional features depicted by X-ray and molecular modelling studies. *Curr. Med. Chem.* 22 783–798. 10.2174/0929867321999141212131457 25312208

[B51] Gudipaty L. Munetz J. Verhoef P. A. Dubyak G. R. (2003). Essential role for Ca2+ in regulation of IL-1beta secretion by P2X7 nucleotide receptor in monocytes, macrophages, and HEK-293 cells. *Am. J. Physiol. Cell Physiol.* 285 C286–C299. 10.1152/ajpcell.00070.2003 12660148

[B52] Guo H. Callaway J. B. Ting J. P. (2015). Inflammasomes: mechanism of action, role in disease, and therapeutics. *Nat. Med.* 21 677–687. 10.1038/nm.3893 26121197 PMC4519035

[B53] Hammad H. Lambrecht B. N. (2015). Barrier epithelial cells and the control of type 2 immunity. *Immunity* 43 29–40. 10.1016/j.immuni.2015.07.007 26200011

[B54] Hattori M. Gouaux E. (2012). Molecular mechanism of ATP binding and ion channel activation in P2X receptors. *Nature* 485 207–212. 10.1038/nature11010 22535247 PMC3391165

[B55] Hauser C. Dayer J. M. Jaunin F. de Rochemonteix B. Saurat J. H. (1986). Intracellular epidermal interleukin 1-like factors in the human epidermoid carcinoma cell line A431. *Cell Immunol.* 100 89–96. 10.1016/0008-8749(86)90009-2 3017569

[B56] He W. T. Wan H. Hu L. Chen P. Wang X. Huang Z. (2015). Gasdermin D is an executor of pyroptosis and required for interleukin-1beta secretion. *Cell Res.* 25 1285–1298. 10.1038/cr.2015.139 26611636 PMC4670995

[B57] He Y. Hara H. Nunez G. (2016a). Mechanism and regulation of NLRP3 inflammasome activation. *Trends Biochem. Sci.* 41 1012–1021. 10.1016/j.tibs.2016.09.002 27669650 PMC5123939

[B58] He Y. Zeng M. Y. Yang D. Motro B. Nunez G. (2016b). NEK7 is an essential mediator of NLRP3 activation downstream of potassium efflux. *Nature* 530 354–357. 10.1038/nature16959 26814970 PMC4810788

[B59] Heid M. E. Keyel P. A. Kamga C. Shiva S. Watkins S. C. Salter R. D. (2013). Mitochondrial reactive oxygen species induces NLRP3-dependent lysosomal damage and inflammasome activation. *J. Immunol.* 191 5230–5238. 10.4049/jimmunol.1301490 24089192 PMC3833073

[B60] Hogquist K. A. Nett M. A. Unanue E. R. Chaplin D. D. (1991). Interleukin 1 is processed and released during apoptosis. *Proc. Natl. Acad. Sci. U.S.A.* 88 8485–8489. 10.1073/pnas.88.19.8485 1924307 PMC52533

[B61] Idzko M. Ferrari D. Eltzschig H. K. (2014). Nucleotide signalling during inflammation. *Nature* 509 310–317. 10.1038/nature13085 24828189 PMC4222675

[B62] Jacobson K. A. Muller C. E. (2016). Medicinal chemistry of adenosine, P2Y and P2X receptors. *Neuropharmacology* 104 31–49. 10.1016/j.neuropharm.2015.12.001 26686393 PMC4871727

[B63] Janeway C. A. Jr. (2001). How the immune system works to protect the host from infection: a personal view. *Proc. Natl. Acad. Sci. U.S.A.* 98 7461–7468. 10.1073/pnas.131202998 11390983 PMC34691

[B64] Jiang L. H. Baldwin J. M. Roger S. Baldwin S. A. (2013). Insights into the molecular mechanisms underlying Mammalian P2X7 receptor functions and contributions in diseases, revealed by structural modeling and single nucleotide polymorphisms. *Front. Pharmacol.* 4:55 10.3389/fphar.2013.00055 PMC3646254 23675347

[B65] Joosten L. A. Netea M. G. Fantuzzi G. Koenders M. I. Helsen M. M. Sparrer H. (2009). Inflammatory arthritis in caspase 1 gene-deficient mice: contribution of proteinase 3 to caspase 1-independent production of bioactive interleukin-1beta. *Arthritis Rheum.* 60 3651–3662. 10.1002/art.25006 19950280 PMC2993325

[B66] Junger W. G. (2011). Immune cell regulation by autocrine purinergic signalling. *Nat. Rev. Immunol.* 11 201–212. 10.1038/nri2938 21331080 PMC4209705

[B67] Kampschmidt R. F. Pulliam L. A. Upchurch H. F. (1973). Sources of leukocytic endogenous mediator in the rat. *Proc. Soc. Exp. Biol. Med.* 144 882–886. 10.3181/00379727-144-37703 4148803

[B68] Karasawa A. Kawate T. (2016). Structural basis for subtype-specific inhibition of the P2X7 receptor. *Elife* 5:e22153 10.7554/eLife.22153 PMC5176352 27935479

[B69] Karmakar M. Katsnelson M. A. Dubyak G. R. Pearlman E. (2016). Neutrophil P2X7 receptors mediate NLRP3 inflammasome-dependent IL-1beta secretion in response to ATP. *Nat. Commun.* 7:10555 10.1038/ncomms10555 PMC4756306 26877061

[B70] Katsnelson M. A. Rucker L. G. Russo H. M. Dubyak G. R. (2015). K+ efflux agonists induce NLRP3 inflammasome activation independently of Ca2+ signaling. *J. Immunol.* 194 3937–3952. 10.4049/jimmunol.1402658 25762778 PMC4390495

[B71] Kawate T. Michel J. C. Birdsong W. T. Gouaux E. (2009). Crystal structure of the ATP-gated P2X(4) ion channel in the closed state. *Nature* 460 592–598. 10.1038/nature08198 19641588 PMC2720809

[B72] Kayagaki N. Stowe I. B. Lee B. L. O’Rourke K. Anderson K. Warming S. (2015). Caspase-11 cleaves gasdermin D for non-canonical inflammasome signalling. *Nature* 526 666–671. 10.1038/nature15541 26375259

[B73] Kepp O. Senovilla L. Vitale I. Vacchelli E. Adjemian S. Agostinis P. (2014). Consensus guidelines for the detection of immunogenic cell death. *Oncoimmunology* 3:e955691 10.4161/21624011.2014.955691 PMC4292729 25941621

[B74] Kim Y. K. Shin J. S. Nahm M. H. (2016). NOD-like receptors in infection, immunity, and diseases. *Yonsei Med. J.* 57 5–14. 10.3349/ymj.2016.57.1.5 26632377 PMC4696971

[B75] Lomedico P. T. Gubler U. Hellmann C. P. Dukovich M. Giri J. G. Pan Y. C. (1984). Cloning and expression of murine interleukin-1 cDNA in *Escherichia coli*. *Nature* 312 458–462. 10.1038/312458a0 6209582

[B76] MacKenzie A. Wilson H. L. Kiss-Toth E. Dower S. K. North R. A. Surprenant A. (2001). Rapid secretion of interleukin-1beta by microvesicle shedding. *Immunity* 15 825–835. 10.1016/S1074-7613(01)00229-1 11728343

[B77] Mansoor S. E. Lu W. Oosterheert W. Shekhar M. Tajkhorshid E. Gouaux E. (2016). X-ray structures define human P2X3 receptor gating cycle and antagonist action. *Nature* 538 66–71. 10.1038/nature19367 27626375 PMC5161641

[B78] Mariathasan S. Newton K. Monack D. M. Vucic D. French D. M. Lee W. P. (2004). Differential activation of the inflammasome by caspase-1 adaptors ASC and Ipaf. *Nature* 430 213–218. 10.1038/nature02664 15190255

[B79] Mariathasan S. Weiss D. S. Newton K. McBride J. O’Rourke K. Roose-Girma M. (2006). Cryopyrin activates the inflammasome in response to toxins and ATP. *Nature* 440 228–232. 10.1038/nature04515 16407890

[B80] Martinon F. Burns K. Tschopp J. (2002). The inflammasome: a molecular platform triggering activation of inflammatory caspases and processing of proIL-beta. *Mol. Cell.* 10 417–426. 10.1016/S1097-2765(02)00599-3 12191486

[B81] Martin-Sanchez F. Diamond C. Zeitler M. Gomez A. I. Baroja-Mazo A. Bagnall J. (2016). Inflammasome-dependent IL-1beta release depends upon membrane permeabilisation. *Cell Death Differ.* 23 1219–1231. 10.1038/cdd.2015.176 26868913 PMC4946890

[B82] Menkin V. (1943). Studies on the isolation of the factor responsible for tissue injury in inflammation. *Science* 97 165–167. 10.1126/science.97.2511.165 17757843

[B83] Minkiewicz J. de Rivero Vaccari J. P. Keane R. W. (2013). Human astrocytes express a novel NLRP2 inflammasome. *Glia* 61 1113–1121. 10.1002/glia.22499 23625868

[B84] Munoz-Planillo R. Kuffa P. Martinez-Colon G. Smith B. L. Rajendiran T. M. Nunez G. (2013). K(+) efflux is the common trigger of NLRP3 inflammasome activation by bacterial toxins and particulate matter. *Immunity* 38 1142–1153. 10.1016/j.immuni.2013.05.016 23809161 PMC3730833

[B85] Murgia M. Hanau S. Pizzo P. Rippa M. Di Virgilio F. (1993). Oxidized ATP. An irreversible inhibitor of the macrophage purinergic P2Z receptor. *J. Biol. Chem.* 268 8199–8203. 8463330

[B86] Nakahira K. Haspel J. A. Rathinam V. A. Lee S. J. Dolinay T. Lam H. C. (2011). Autophagy proteins regulate innate immune responses by inhibiting the release of mitochondrial DNA mediated by the NALP3 inflammasome. *Nat. Immunol.* 12 222–230. 10.1038/ni.1980 21151103 PMC3079381

[B87] Nie Y. Yang D. Oppenheim J. J. (2016). Alarmins and antitumor immunity. *Clin. Ther.* 38 1042–1053. 10.1016/j.clinthera.2016.03.021 27101817 PMC6314656

[B88] North R. A. (2016). P2X receptors. *Philos. Trans. R. Soc. Lond. B Biol. Sci.* 371 20150427 10.1098/rstb.2015.0427 PMC4938027 27377721

[B89] Ogura Y. Sutterwala F. S. Flavell R. A. (2006). The inflammasome: first line of the immune response to cell stress. *Cell* 126 659–662. 10.1016/j.cell.2006.08.002 16923387

[B90] Pelegrin P. Barroso-Gutierrez C. Surprenant A. (2008). P2X7 receptor differentially couples to distinct release pathways for IL-1beta in mouse macrophage. *J. Immunol.* 180 7147–7157. 10.4049/jimmunol.180.11.7147 18490713

[B91] Pelegrin P. Surprenant A. (2007). Pannexin-1 couples to maitotoxin- and nigericin-induced interleukin-1beta release through a dye uptake-independent pathway. *J. Biol. Chem.* 282 2386–2394. 10.1074/jbc.M610351200 17121814

[B92] Pellegatti P. Falzoni S. Pinton P. Rizzuto R. Di Virgilio F. (2005). A novel recombinant plasma membrane-targeted luciferase reveals a new pathway for ATP secretion. *Mol. Biol. Cell* 16 3659–3665. 10.1091/mbc.E05-03-0222 15944221 PMC1182305

[B93] Perregaux D. Barberia J. Lanzetti A. J. Geoghegan K. F. Carty T. J. Gabel C. A. (1992). IL-1 beta maturation: evidence that mature cytokine formation can be induced specifically by nigericin. *J. Immunol.* 149 1294–1303. 1500719

[B94] Perregaux D. Gabel C. A. (1994). Interleukin-1 beta maturation and release in response to ATP and nigericin. Evidence that potassium depletion mediated by these agents is a necessary and common feature of their activity. *J. Biol. Chem.* 269 15195–15203. 8195155

[B95] Pizzirani C. Ferrari D. Chiozzi P. Adinolfi E. Sandona D. Savaglio E. (2007). Stimulation of P2 receptors causes release of IL-1beta-loaded microvesicles from human dendritic cells. *Blood* 109 3856–3864. 10.1182/blood-2005-06-031377 17192399

[B96] Prochnicki T. Mangan M. S. Latz E. (2016). Recent insights into the molecular mechanisms of the NLRP3 inflammasome activation. *F1000Res* 5:F1000 10.12688/f1000research.8614.1 PMC4963208 27508077

[B97] Qu Y. Franchi L. Nunez G. Dubyak G. R. (2007). Nonclassical IL-1 beta secretion stimulated by P2X7 receptors is dependent on inflammasome activation and correlated with exosome release in murine macrophages. *J. Immunol.* 179 1913–1925. 10.4049/jimmunol.179.3.1913 17641058

[B98] Qu Y. Ramachandra L. Mohr S. Franchi L. Harding C. V. Nunez G. (2009). P2X7 receptor-stimulated secretion of MHC class II-containing exosomes requires the ASC/NLRP3 inflammasome but is independent of caspase-1. *J. Immunol.* 182 5052–5062. 10.4049/jimmunol.0802968 19342685 PMC2768485

[B99] Rada B. Park J. J. Sil P. Geiszt M. Leto T. L. (2014). NLRP3 inflammasome activation and interleukin-1beta release in macrophages require calcium but are independent of calcium-activated NADPH oxidases. *Inflamm. Res.* 63 821–830. 10.1007/s00011-014-0756-y 25048991 PMC4162906

[B100] Ramachandra L. Qu Y. Wang Y. Lewis C. J. Cobb B. A. Takatsu K. (2010). Mycobacterium tuberculosis synergizes with ATP to induce release of microvesicles and exosomes containing major histocompatibility complex class II molecules capable of antigen presentation. *Infect. Immun.* 78 5116–5125. 10.1128/IAI.01089-09 20837713 PMC2981298

[B101] Rathinam V. A. Fitzgerald K. A. (2016). Inflammasome complexes: emerging mechanisms and effector functions. *Cell* 165 792–800. 10.1016/j.cell.2016.03.046 27153493 PMC5503689

[B102] Rubartelli A. Cozzolino F. Talio M. Sitia R. (1990). A novel secretory pathway for interleukin-1 beta, a protein lacking a signal sequence. *EMBO J.* 9 1503–1510. 2328723 10.1002/j.1460-2075.1990.tb08268.x PMC551842

[B103] Salaro E. Rambaldi A. Falzoni S. Amoroso F. S. Franceschini A. Sarti A. C. (2016). Involvement of the P2X7-NLRP3 axis in leukemic cell proliferation and death. *Sci. Rep.* 6:26280 10.1038/srep26280 PMC4879576 27221966

[B104] Sanman L. E. van der Linden W. A. Verdoes M. Bogyo M. (2016). Bifunctional probes of cathepsin protease activity and pH reveal alterations in endolysosomal pH during bacterial infection. *Cell Chem. Biol.* 23 793–804. 10.1016/j.chembiol.2016.05.019 27427229 PMC4982764

[B105] Sanz J. M. Chiozzi P. Ferrari D. Colaianna M. Idzko M. Falzoni S. (2009). Activation of microglia by amyloid {beta} requires P2X7 receptor expression. *J. Immunol.* 182 4378–4385. 10.4049/jimmunol.0803612 19299738

[B106] Schroder K. Tschopp J. (2010). The inflammasomes. *Cell* 140 821–832. 10.1016/j.cell.2010.01.040 20303873

[B107] Shi J. Zhao Y. Wang K. Shi X. Wang Y. Huang H. (2015). Cleavage of GSDMD by inflammatory caspases determines pyroptotic cell death. *Nature* 526 660–665. 10.1038/nature15514 26375003

[B108] Shi J. Zhao Y. Wang Y. Gao W. Ding J. Li P. (2014). Inflammatory caspases are innate immune receptors for intracellular LPS. *Nature* 514 187–192. 10.1038/nature13683 25119034

[B109] Shimada K. Crother T. R. Karlin J. Dagvadorj J. Chiba N. Chen S. (2012). Oxidized mitochondrial DNA activates the NLRP3 inflammasome during apoptosis. *Immunity* 36 401–414. 10.1016/j.immuni.2012.01.009 22342844 PMC3312986

[B110] Sneddon P. Westfall D. P. (1984). Pharmacological evidence that adenosine triphosphate and noradrenaline are co-transmitters in the guinea-pig vas deferens. *J. Physiol.* 347 561–580. 10.1113/jphysiol.1984.sp015083 6142947 PMC1199464

[B111] Solle M. Labasi J. Perregaux D. G. Stam E. Petrushova N. Koller B. H. (2001). Altered cytokine production in mice lacking P2X(7) receptors. *J. Biol. Chem.* 276 125–132. 10.1074/jbc.M006781200 11016935

[B112] Suadicani S. O. Brosnan C. F. Scemes E. (2006). P2X7 receptors mediate ATP release and amplification of astrocytic intercellular Ca2+ signaling. *J. Neurosci.* 26 1378–1385. 10.1523/JNEUROSCI.3902-05.2006 16452661 PMC2586295

[B113] Surprenant A. Rassendren F. Kawashima E. North R. A. Buell G. (1996). The cytolytic P2Z receptor for extracellular ATP identified as a P2X receptor (P2X7). *Science* 272 735–738. 10.1126/science.272.5262.735 8614837

[B114] Thornberry N. A. Bull H. G. Calaycay J. R. Chapman K. T. Howard A. D. Kostura M. J. (1992). A novel heterodimeric cysteine protease is required for interleukin-1 beta processing in monocytes. *Nature* 356 768–774. 10.1038/356768a0 1574116

[B115] Turola E. Furlan R. Bianco F. Matteoli M. Verderio C. (2012). Microglial microvesicle secretion and intercellular signaling. *Front. Physiol.* 3:149 10.3389/fphys.2012.00149 PMC3357554 22661954

[B116] Venereau E. Ceriotti C. Bianchi M. E. (2015). DAMPs from cell death to new life. *Front. Immunol.* 6:422 10.3389/fimmu.2015.00422 PMC4539554 26347745

[B117] Verderio C. Muzio L. Turola E. Bergami A. Novellino L. Ruffini F. (2012). Myeloid microvesicles are a marker and therapeutic target for neuroinflammation. *Ann. Neurol.* 72 610–624. 10.1002/ana.23627 23109155

[B118] Vigano E. Diamond C. E. Spreafico R. Balachander A. Sobota R. M. Mortellaro A. (2015). Human caspase-4 and caspase-5 regulate the one-step non-canonical inflammasome activation in monocytes. *Nat. Commun.* 6:8761 10.1038/ncomms9761 PMC4640152 26508369

[B119] Vince J. E. Silke J. (2016). The intersection of cell death and inflammasome activation. *Cell Mol. Life Sci.* 73 2349–2367. 10.1007/s00018-016-2205-2 27066895 PMC11108284

[B120] Walev I. Reske K. Palmer M. Valeva A. Bhakdi S. (1995). Potassium-inhibited processing of IL-1 beta in human monocytes. *EMBO J.* 14 1607–1614. 7737113 10.1002/j.1460-2075.1995.tb07149.x PMC398253

[B121] Wang Y. Martins I. Ma Y. Kepp O. Galluzzi L. Kroemer G. (2013). Autophagy-dependent ATP release from dying cells via lysosomal exocytosis. *Autophagy* 9 1624–1625. 10.4161/auto.25873 23989612

[B122] Wewers M. D. (2004). IL-1beta: an endosomal exit. *Proc. Natl. Acad. Sci. U.S.A.* 101 10241–10242. 10.1073/pnas.0403971101 15240873 PMC478557

[B123] Yang D. He Y. Munoz-Planillo R. Liu Q. Nunez G. (2015). Caspase-11 requires the pannexin-1 channel and the purinergic P2X7 pore to mediate pyroptosis and endotoxic shock. *Immunity* 43 923–932. 10.1016/j.immuni.2015.10.009 26572062 PMC4795157

[B124] Zhou R. Yazdi A. S. Menu P. Tschopp J. (2011). A role for mitochondria in NLRP3 inflammasome activation. *Nature* 469 221–225. 10.1038/nature09663 21124315

